# Advances in Parkinson’s Disease: 200 Years Later

**DOI:** 10.3389/fnana.2018.00113

**Published:** 2018-12-14

**Authors:** Natalia López-González Del Rey, Ana Quiroga-Varela, Elisa Garbayo, Iria Carballo-Carbajal, Rubén Fernández-Santiago, Mariana H. G. Monje, Inés Trigo-Damas, María J. Blanco-Prieto, Javier Blesa

**Affiliations:** ^1^HM CINAC, Hospital Universitario HM Puerta del Sur, Madrid, Spain; ^2^Biomedical Research Networking Center on Neurodegenerative Diseases (CIBERNED), Madrid, Spain; ^3^Department of Neuroscience, Centro de Investigación Médica Aplicada (CIMA), University of Navarra, Pamplona, Spain; ^4^Pharmaceutical Technology and Chemistry, School of Pharmacy and Nutrition, University of Navarra, Pamplona, Spain; ^5^Instituto de Investigación Sanitaria de Navarra (IdiSNA), Pamplona, Spain; ^6^Neurodegenerative Diseases Research Group, Vall d’Hebron Research Institute, Barcelona, Spain; ^7^Laboratory of Parkinson Disease and other Neurodegenerative Movement Disorders, Department of Neurology, Hospital Clínic de Barcelona, Institut d’Investigacions Biomèdiques August Pi i Sunyer (IDIBAPS), University of Barcelona, Barcelona, Spain; ^8^Department of Anatomy, Histology and Neuroscience, Facultad de Medicina, Universidad Autónoma de Madrid, Madrid, Spain

**Keywords:** Parkinson’s disease, genetics, drug delivery systems, non-motor symptoms, focused ultrasound

## Abstract

When James Parkinson described the classical symptoms of the disease he could hardly foresee the evolution of our understanding over the next two hundred years. Nowadays, Parkinson’s disease is considered a complex multifactorial disease in which genetic factors, either causative or susceptibility variants, unknown environmental cues, and the potential interaction of both could ultimately trigger the pathology. Noteworthy advances have been made in different fields from the clinical phenotype to the decoding of some potential neuropathological features, among which are the fields of genetics, drug discovery or biomaterials for drug delivery, which, though recent in origin, have evolved swiftly to become the basis of research into the disease today. In this review, we highlight some of the key advances in the field over the past two centuries and discuss the current challenges focusing on exciting new research developments likely to come in the next few years. Also, the importance of pre-motor symptoms and early diagnosis in the search for more effective therapeutic options is discussed.

## A Little Bit of History

Two centuries have passed since James Parkinson’s *Essay on the Shaking Palsy* described a handful of patients who showed tremor at rest, bradykinesia and, some of them, akinesia. In his essay, he characterized the motor symptoms of the disease that now takes his name (Parkinson, 1817). Although this was the first description of the disease as a neurological condition, it was not until 50 years later that new scientific evidence obtained by Jean-Martin Charcot contributed to a definition of the clinical and anatomopathological basis of Parkinson’s Disease (PD) (Charcot, 1872). Years later, in 1893, Blocq and Marinescu noticed resting tremor in a patient that resembled parkinsonian symptoms. The tremor was due to a tuberculous granuloma on the right cerebral peduncle that was affecting the ipsilateral *Substantia nigra pars compacta* (SNc) (Blocq and Marinescu, 1893). It was Brissaud a few years later who suggested that the SNc might be the site affected in PD (Brissaud, 1899). Two decades later, Trétiakoff first reported neuropathological changes in the SNc in PD patients. He observed a large loss of neuromelanin in the SNc resulting from the absence of SNc neurons containing this pigment and also the presence of cytoplasmatic inclusions named Lewy bodies (LB) (Trétiakoff, 1919). These structures had been described some years earlier by James Lewy and, from that moment onward, this feature of PD became the focal point of neuropathological studies on PD (Lewy, 1912). The presence of both loss of dopaminergic neurons in the SNc and LB was established as the anatomopathological hallmark and diagnostic criterion of PD (Postuma and Berg, 2016).

At this point, the diagnostic criteria were established, but the main challenge was to assure successful treatment. The first neurosurgery of the basal ganglia (BG) to treat PD took place in 1940. Between the late 1950s and the mid-1960s many discoveries were made about the existence of dopamine (DA) as a neurotransmitter (Montagu, 1957; Carlsson et al., 1958) and its role in the striatum (Bertler and Rosengren, 1959; Carlsson, 1959; Sano et al., 1959). In 1957, Carlsson reported the first evidence for a functional role for DA, describing the reserpine effect in reducing motor activity in animals, which was reversed by L-3,4-dihydroxyphenylalanine (L-DOPA) administration, a precursor in DA synthesis. DA signaling proved to play a crucial role in motor control by the BG (Carlsson et al., 1957). Soon after this, evidence emerged of the striatal DA deficiency in PD (Sano, 2000). Particularly, Ehringer and Hornykiewicz described a deficit in both the striatum and the SNc in brains from parkinsonian patients (Ehringer and Hornykiewicz, 1960). Furthermore, some studies sustained the presence of a dopaminergic nigrostriatal projections and they also revealed that the dorsolateral striatum mainly receives terminals from SNc neurons. It happens that this area of the striatum is the most affected in PD (Dahlstroem and Fuxe, 1964; Anden et al., 1965). After these discoveries, the L-DOPA era began. During these years, it was demonstrated that intravenous injection of L-DOPA and also small oral doses of L-DOPA in humans had anti parkinsonian effects (Cotzias, 1968). From that moment L-DOPA became the gold-standard treatment for PD, since many authors consistently reported a marked improvement in PD with large oral doses of L-DOPA (Hornykiewicz, 2002). Since then significant progress has been made in the development of new pharmacological and surgical tools to treat PD motor symptoms (Smith et al., 2012).

A new important breakthrough took place in 1983 when Langston and colleagues reported a group of drug users who developed acute parkinsonism after MPTP (1-methyl-4-phenyl-1,2,3,6-tetrahydropyridine) exposure (Langston et al., 1983). These patients developed an acute syndrome indistinguishable from PD. This is due because the MPTP metabolite, MPP+, destroys the dopaminergic neurons in the substantia nigra after a series of alterations in the mitochondrial matrix and the electron transport chain. The SNc of Parkinson patients was also described as exhibiting a marked decrease in complex I activity (Davis et al., 1979; Schapira, 1993). The fact that some PD patients have certain polymorphisms in genes that express subunits of complex I suggests that this could be a vulnerability factor in PD (Kösel et al., 1998; van der Walt et al., 2003). New models based on MPTP intoxication allowed researchers to ascertain PD hallmarks both *in vitro* and *in vivo* (Langston, 2017). Due to the achievements of pharmacological DA treatments, search of cell-based DA replacement approaches were initiated with largely disappointing results (Barker et al., 2015). From the surgical and therapeutic point of view, discrete lesions of the BG improved parkinsonism (Meyers, 1942). A monkey model of PD showed motor signs improvement as a result of the chemical destruction of the subthalamic nucleus (STN) (Bergman et al., 1990), with evidence of reversal of experimental parkinsonism by STN lesions. This same year deep brain stimulation (DBS) of the STN became effective for PD treatment (Hammond et al., 2007; Rosin et al., 2011; de Hemptinne et al., 2015).

In the late 1990s, due to the improvement and sophistication of genetic analysis techniques, mutations in *SNCA* gene codifying for alpha-synuclein (α-syn) protein were identified as the first genetic cause of PD (Polymeropoulos et al., 1997). Once it was clear that *SNCA* mutations cause parkinsonism, and more than 80 years after the discovery of LB, α-syn protein was found to be the main component of LB (Spillantini et al., 1997, 1998). Later on and based on these discoveries, Braak et al. (2003) proposed a pathological staging of the disease. From that time on, genetic studies have revealed many other mutations in other genes related to PD (*PINK1, LRRK2, Parkin, DJ1*, etc… see *Advances in genetics* below). The discovery of different genetic variants affecting the risk of PD has provided the field with a new battery of potential therapies ready to be tested in clinical trials. The initial findings have been followed by intensive research and the identification of several genes linked to PD pathogenesis in the last few years. Developments in genetics and molecular techniques such as CRISPR, have allowed the raise of new experimental models based on the use of transgenic animals presenting mutations associated to PD (Trigo-Damas et al., 2018). These examples open the door to studies where using genetic-based animal models would allow to assess the potential role of α-syn aggregation and spreading, evaluating potential therapeutics, imaging tracers or biomarkers (Dehay et al., 2016; Koprich et al., 2017; Ko and Bezard, 2017; Marmion and Kordower, 2018); also, the cellular models offer unique opportunities for the identification of therapeutic strategies capable of modulating the disease (Lázaro et al., 2017). These new models join the well-known classic neurotoxin based animal models such as MPTP or 6-OHDA that have provided a valuable insight into potential new targets for disease intervention (Blesa et al., 2012; Morissette and Di Paolo, 2018).

**FIGURE 1:**
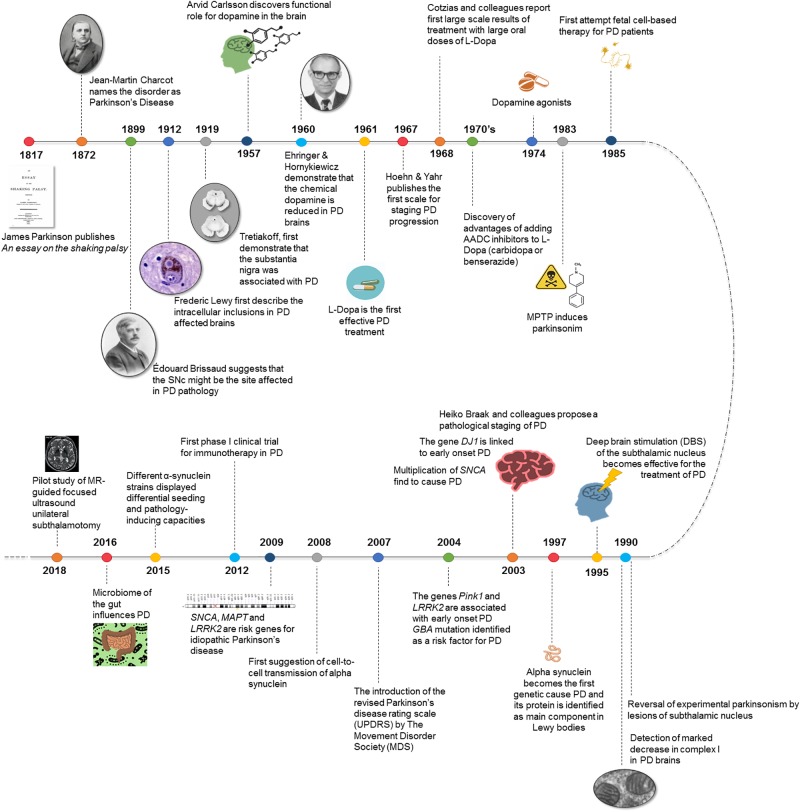
Breakthroughs in Parkinson’s disease history.

At present, catching theories linking alterations in the gut microbiota to PD of the disease open new research areas in the hunt of the etiology of the disease (Sampson et al., 2016). Many efforts are concentrated in decoding the pre-symptomatic phases and turning scientific progresses into disease-modifying therapies for PD (Blesa et al., 2017). In this sense, exciting cutting-edge approaches with less invasive technologies such as gamma knife or focused ultrasound for the treatment of motor symptoms in PD have been advanced (Martínez-Fernández et al., 2018) (see *New technologies for the diagnosis, clinical assessment and treatment of Parkinson’s Disease* below) (Figure 1).

## Advances in Genetics

The etiology of PD remains largely unknown. The majority of patients are classified as idiopathic PD cases, i.e., arising ‘spontaneously’ or from an unknown cause. Prevalence is estimated at 1% in people above 65 years and increases exponentially in subsequent decades of life (Fahn, 2003) and in fact, aging is considered the major known risk factor. Yet, continuous and intense efforts have been undertaken to improve our incomplete comprehension of the disease. In this context, genetic research has played a pivotal role in elucidating the cause of disease, most especially during the last 20 years. Earlier than that, the genetic contribution to PD was unrecognized because classic reports of PD familial clustering or twin concordance studies were scarce and controversial (Farrer, 2006). It was in 1997 when a linkage study first identified unequivocal familial segregation of the missense mutation A53T in the *SNCA* gene with an adult-onset autosomal-dominant PD phenotype (Polymeropoulos et al., 1997). Subsequently, other pathogenic missense mutations in *SNCA* were identified including A30P, E46K, H50Q, G51N, and A53T (Krüger et al., 1998; Zarranz et al., 2004; Lesage et al., 2013; Proukakis et al., 2013). In 1998, another pioneer study reported that the α-synuclein protein was the main component of the proteinaceous aggregates termed Lewy bodies and Lewy neurites which are found in the soma and neurites of the few surviving dopaminergic neurons of the SNpc in PD patients (Spillantini et al., 1997, 1998). The identification of *SNCA* led to a shift of paradigm in the classification of PD patients into monogenic or familial PD (fPD) cases caused by pathogenic mutations in genes associated with the disease (5–10% of cases), and the vast majority of patients encompassing sporadic PD (sPD) cases (95%). The identification of SNCA was also seminal to set the basis for the subsequent intense genetic cell and animal modeling of the disease in the lab (Singleton et al., 2003; Chartier-Harlin et al., 2004; Ibáñez et al., 2004). More recently, multiplications of the *SNCA* locus, duplications and triplications, were found to cause PD with an inverse correlation between gene dose and age-at-onset, but a direct effect on disease severity (Chartier-Harlin et al., 2004; Ibáñez et al., 2004; Singleton et al., 2013). Overall mutations in *SNCA* are uncommon in frequency and lead to a DOPA-responsive early-onset parkinsonism, often severe and with dementia that is pathologically characterized by nigral neurodegeneration and widespread brainstem and cortical LB pathology.

Until date, a total of 23 loci and 19 causative genes have been associated with PD, yet with certain degree of heterogeneity regarding phenotypes (PD only or PD plus syndromes), age-at-onset (juvenile or adult onset), and inheritance mode (autosomal dominant, recessive or X-linked) (Table 1). Whereas some of the genes associated to the *PARK* loci have not been yet identified (*PARK3, PARK10, PARK12*, and *PARK16*), the pathogenicity of a few PD-associated genes still remains controversial due to novelty or to lack of replication of the original study (*UCHL1, GIGYF2, EIF4G1, SYNJ1, TMEM230*, and *CHCHD2*). Yet, mutations in the remaining genes, although rare in frequency, have been unequivocally established as PD-causative and account for the majority of autosomal dominant (*SNCA*, *LRRK2*, *HTRA2*, and *VPS35*) or recessive PD cases (*PRKN*, *PINK1*, *DJ-1, ATP13A2, PLA2G6, FBXO7, DNAJC6*, and *VPS13C*). Among the dominant genes, the identification by linkage analysis of mutations in the leucine-rich repeat gene (*LRRK2*) in some PD families with adult onset autosomal-dominant inheritance simultaneously by two groups (Paisan-Ruiz et al., 2004; Zimprich et al., 2004) represented another major milestone in PD research. Subsequently, three different groups identified in parallel the mutation G2019S at the kinase domain of LRRK2 as the most common pathogenic variant of *LRRK2-*associated PD (Di Fonzo et al., 2005; Hernandez et al., 2005; Kachergus et al., 2005) that remarkably it is found not only in monogenic but also in sPD cases lacking mendelian segregation. The *LRRK2*-associated PD form uniquely resembles common sPD at the clinical and neuropathological levels, yet with slight clinical differences (Marras et al., 2016; Pont-Sunyer et al., 2017), and eventual pleomorphic pathology (Zimprich et al., 2004). Of note, the penetrance of G2019S mutation is limited but rises progressively with age (Healy et al., 2008; Marder et al., 2015) and has been shown to be modified by additional factors such as genetic risk polymorphisms and other still unknown factors (Trinh et al., 2016; Fernández-Santiago et al., 2018). Moreover, mutations in HtrA Serine Peptidase 2 (*HTRA2*) (Strauss et al., 2005) and vesicle protein sorting 35 (*VPS35*) (Vilariño-Güell et al., 2011; Zimprich et al., 2011) are responsible of typical L-DOPA responsive PD, although no neuropathological data is not available yet. On the other hand, mutations in the recessive genes including parkin (*PRKN*), PTEN-induced putative kinase 1 (*PINK1*) and *DJ-1* are causative of early-onset parkinsonism shearing largely identical clinical phenotypes, but distinct neuropathology. *PRKN*-associated PD is characterized by pure degeneration in the *SNc* and *locus coeruleus* without LB pathology and occasional Tau inclusions (Schneider and Alcalay, 2017), whereas *PINK1* mutations lead to nigral neurodegeneration with LB and neurites (Samaranch et al., 2010), and *DJ1*-associated pathology includes severe degeneration in the SNc and *locus coeruleus* with diffuse LBs and axonal spheroids (Taipa et al., 2016). In addition, pathogenic mutations in the genes ATPase 13A2 (*ATP13A2*) (Bras et al., 2012), phospholipase A2 (*PLA2G6*) (Gregory et al., 2008) F-Box protein 7 (*FBXO7*) (Shojaee et al., 2008), DNA J Heat Shock Protein Family (Hsp40) Member C6 (*DNAJC6*) (Edvardson et al., 2012) and Vacuolar Protein Sorting 13 Homolog C (*VPS13C*) (Lesage et al., 2016) are linked to autosomal recessive, early-onset atypical parkinsonism that often comprises additional clinical features such as pyramidal degeneration, ataxia or dementia, with or without LBs. Overall, although the relative contribution of pathogenic mendelian genes to overall PD is limited, genetic research in PD has been instrumental since it has uniquely permitted the identification of disease molecular alterations, pathophysiological pathways, and candidate therapeutic targets, most of which are believed to be largely common to sPD. Thus genetic findings in PD have undoubtly paved the way out for tackling overall pathology of all PD cases.

**Table 1:** Summary of genes associated with Parkinson’s disease.

*Locus*	Gene	Inheritance	Onset	Location	Variants	Function
*PARK1/4*	*SNCA*	Dominant Risk factor	EO	4q21.3-q22	5 point mutations, multiplications Rep1 risk variant in the promoter	Synaptic vesicles trafficking
*PARK2*	*PARKIN*	Recessive	EO	6q25.2-q27	>250 point mutation, ins/de and exon rearrangements	Mitophagy
*PARK3*	Unknown	Dominant	LO	2p13	?	?
*PARK5*	*UCHL1*	Dominant	LO	4p13	1 missense variant in one sibling pair	Proteasome
*PARK6*	*PINK1*	Recessive	EO	1p36.12	>100 point mutations, ins/del and exon rearrangements	Mitophagy
*PARK7*	*DJ-1*	Recessive	EO	1p36.23	>20 point mutations and deletions	Mitophagy
*PARK8*	*LRRK2*	Dominant	LO	12q12	7 point mutations	Autophagy?
		Risk factor			Risk variants p.R1628P and p.G2385R	
*PARK9*	*ATP13A2*	Recessive	EO	1p36	>20 point mutations	Lysosomes
*PARK10*	Unknown	Risk factor	?	1p32	?	?
*PARK11*	*GIGYF2*	Recessive	EO	2q36-7	7 missense variants	Insulin-like growth factors (IGFs) signaling
*PARK12*	Unknown	Risk factor	?	Xq21-q22	?	?
*PARK13*	*HTRA2*	Dominant	?	2p13.1	1 missense variant	Mitophagy,
*PARK14*	*PLA2G6*	Recessive	EO	22q13.1	>18 missense variants	Lipids metabolism
*PARK15*	*FBXO7*	Recessive	EO	22q12.3	4 point mutations	Mitophagy
*PARK16*	Unknown	Risk factor	?	1q32	?	?
*PARK17*	*VPS35*	Dominant	LO	16q12	2 point mutations	Endosomes
*PARK18*	*EIF4G1*	Dominant	LO	3q27.1	1 missense variant	Protein translation
*PARK19*	*DNAJC6*	Recessive	EO	1p31.3	9 missense variants	Endosomes
*PARK20*	*SYNJ1*	Recessive	EC	21q22.11	3 missense variants	Endosomes
*PARK21*	*DNAJC13*	Dominant	LO	3q22.1	1 missense variant	Endosomes
*PARK22*	*CHCHD2*	Dominant	LO/EO	7p11.2	1 missense variant, 1 truncation	Mitochondria-mediated apoptosis and metabolism?
*PARK23*	*VPS13C*	Recessive	EO	15q22.2	2 missense variants,l truncation	Mitophagy
–	*GBA*	AD, AR in GD Risk factor	LO	lq22	>10 missense variants	Lysosomes
–	*MAPT*	Sporadic Risk factor		17q21.31	H1 haplotype increase PD risk and disease severity	Microtubules


*EO, early onset; LO, late onset.*

In addition to PD-causative mutations, classical candidate gene association approaches or more recently large genome-wide association studies (GWAS) have identified common genetic variants in genes such as *SNCA*, *LRRK2*, microtubule-associated protein tau gene (*MAPT*) or glucosylceramidase beta (*GBA*) which contribute to increase PD susceptibility (Lill, 2016). The variants in *MAPT* (Pastor et al., 2000; Golbe et al., 2001; Caffrey and Wade-Martins, 2007) and *SNCA* (Botta-Orfila et al., 2011; Cardo et al., 2012; Brockmann et al., 2013) loci showed the strongest association with PD risk across populations and most importantly, also at the GWAS level (Simón-Sánchez et al., 2009; Bonifati, 2010; Nalls et al., 2014) correlating not only with higher risk but with increased disease age-at-onset as well (Wang G. et al., 2016). On the other hand, common variants in LRRK2 increase the risk of PD only in Asian populations but not in Europeans (Farrer et al., 2007; Lu et al., 2008). In addition, mutations in *GBA* which codifies the lysosomal enzyme β-glucocerebrosidase are causative of the recessive lysosomal storage disorder Gaucher’s disease. Both homozygous and heterozygous *GBA* variants increase the risk of developing PD (Thaler et al., 2017). Moreover, *GBA*-mutation carriers show a more severe parkinsonism than idiopathic patients, earlier age-at-onset and more frequently dementia (Thaler et al., 2017). Besides genetics, epigenetic alterations have also been suggested to play a role in the pathogenesis of PD in recent years. Thus, abnormal changes in various epigenetic mechanisms regulating gene expression such as DNA methylation (Masliah et al., 2013; Coupland et al., 2014; Fernández-Santiago et al., 2015; Pihlstrom et al., 2015), histones modifications (Park et al., 2016) or microRNAs (miRNAs) (Kim et al., 2007) have been linked to disease, thus opening a new venue of epigenetic research in PD.

## From Motor to Non-Motor Symptoms

Motor disturbances in PD have been widely investigated leading to a better diagnosis and origination of validated rating scales and therapies. However, the non-motor symptoms (NMS) of PD also have major importance when evaluating the quality of life of patients and the impact on health economics, attracting a growing interest in the last years. The incidence of NMS augments along with the disease duration, even preceding the motor symptoms or signs by several years. Symptoms such as olfactory dysfunction, REM sleep behavior disorder (RBD), constipation, depression, and pain (Chaudhuri et al., 2006; Tolosa et al., 2009) appear to be clear indicators of a preclinical phase of the disease. This concept is reinforced by studies showing an augmented risk for patients with idiopathic RBD or idiopathic hyposmia to develop a synucleinopathy (Boeve et al., 2001; Iranzo et al., 2014; Sakakibara et al., 2014; Postuma et al., 2017).

In early phases of the disease, some of these NMS still remain in many patients. Up to 21% of patients report pain, depression or anxiety (O’Sullivan et al., 2008). Importantly, in many cases, patients report a major disturbances of these NMS rather than motor ones, in the beginning of the disease (Gulati et al., 2004; Politis et al., 2010).

We currently know that PD involves disorders in several neurotransmitters pathways, including the cholinergic, noradrenergic, and serotonergic systems (Wolters, 2009; Halliday et al., 2011; Jellinger, 2012; Buddhala et al., 2015). This fact could relate symptoms such as depression with the loss of dopaminergic and noradrenergic transmission in the limbic system; and also anxiety and apathy, associated in this case to the low dopaminergic transmission (Thobois et al., 2010). On the other hand, excess of dopaminergic transmission due to DA agonist therapy, can prompt some NMS. Impulse control disorders (ICDs) is one of the most common side effects of dopamine replacement therapy used in PD with an estimated prevalence of 4.9–19% (Voon et al., 2009; Weintraub et al., 2010). ICDs are behavioral addictions including exaggerated behaviors such as gambling or shopping related to the administration of D2/D3 agonists. Research on this topic remains quite reduced and preclinical studies are limited because of the lack of alternatives for the pharmacological treatment. (Voon et al., 2009). It seems that the fact that in PD-ICD patients there is a major denervation in the ventral striatum this leads to an “over-dose” when dopamine is administered in ventral areas and limbic pathways (Weintraub, 2009; Voon et al., 2011). Nevertheless, further studies should be performed in order to achieve a better comprehension of the disorder and the development of successful treatments.

Non-motor fluctuations (uncomfortable anxiety, slowness of thinking, fatigue, and dysphoria) are other psychiatric disorder also DA-dependent, and reported primarily during “OFF” periods and can be reversed with continuous dopaminergic replacement (Chaudhuri and Schapira, 2009).

Nowadays, NMS denote some of the most relevant sources of disability and impairment in quality of life of parkinsonian patients and the acknowledgment of these symptoms become critical for the improvement and advances in the diagnosis of the disease (Chaudhuri et al., 2006). Still, in many cases, NMS of PD are not distinguished in routine clinical evaluations since their origin are not directly related to PD (Shulman et al., 2002).

These circumstances indicate the relevance of developing successful tools to identify NMS, both for the assessment and for their treatment (Grosset et al., 2007). The development of valuable instruments capable of supporting neurologists at the time of diagnosis would also mean a benefit for the rise of valid therapeutic strategies (Seppi et al., 2011) (see *Diagnosis and clinical assessment devices* below). This is reflected in the scarce therapies available for non-motor deficits (Zesiewicz et al., 2010). Currently, dopaminergic treatments are the most broadly used therapies, but they have no impact on those aspects of the disease that are associated to other neurotransmitter deficits. Conversely, the use of anticholinergics, for example, classically increases the cognitive symptoms of PD, as it does deep brain stimulation surgery (Witt et al., 2008).

To sum up, the increasing prevalence of non-motor complications is far complex marking a new concept in the scenery of PD. These problems are linked with a marked decrease of quality of life of the patients and the social life of their families. Their etiology is multifaceted and still poorly understood. Thus, specific NMS treatments are required, as current treatment options for NMS in PD continue incomplete and large areas remain unfulfilled of therapeutic need.

## New Technologies for the Diagnosis, Clinical Assessment and Treatment of Parkinson’s Disease

In the last decade, new technology-based tools and technology-based therapies have been advanced with the objective of refining the diagnosis, clinical assessment and treatment of patients with movement disorders. The development and intricacy of molecular and cellular techniques, as well as extraordinary progress in technology, have marked a milestone in our general understanding of the disease.

### Drug Delivery Systems

**FIGURE 2:**
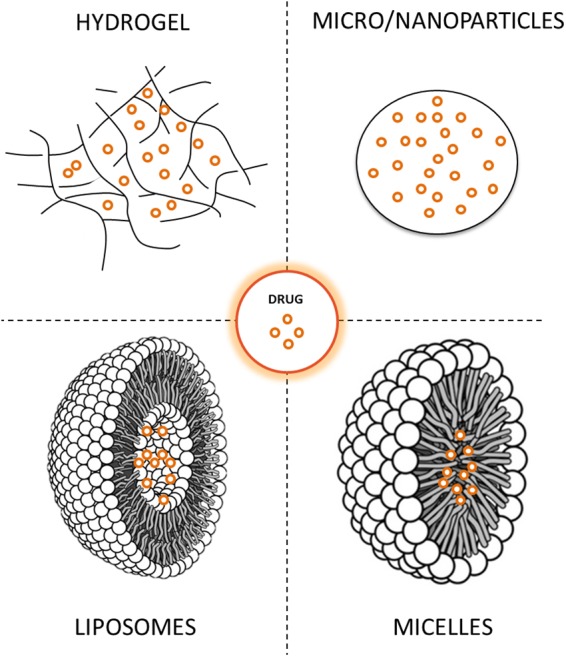
Drug delivery systems under investigation for Parkinson’s disease treatment.

The clinical use of neuroprotective molecules has been hampered by several issues, and among these, drug delivery to the brain remains a particular challenge. To address these limitations, drug delivery systems and methods that allow enhanced brain delivery of neuroprotective molecules have been investigated. These new technologies offer unprecedented advantages enabling protection of sensitive molecules from degradation and controlled release over days or months. Drug delivery systems can also be engineered to target diseased regions within the body, thereby enhancing the specificity of therapeutics. Therefore, the delivery and efficacy of many pharmaceutical compounds can be improved and their side effects reduced. Among drug delivery systems, microparticles (MPs), nanoparticles (NPs) and hydrogels (HGs) seem to be the most effective in providing neuroprotection, although liposomes and micelles have also been investigated (Figure 2) (Garbayo et al., 2009; Rodríguez-Nogales et al., 2016). MPs and NPs are particulate carrier systems in the micrometer and nanometer size range, respectively. MPs are generally used for the long-term delivery of drugs while NPs are commonly used as carriers of small molecules for targeted and intracellular delivery. On the other hand, HGs are tridimensional polymeric networks that absorb a large amount of water, which becomes their principal component. Formulations can be designed either for local administration into the brain or for systemic delivery to achieve targeted action in the central nervous system. The examples below show that drug delivery systems are in the initial stages of the drug development process, but the potential for using this technology for PD treatment is very high.

### Drug Delivery Systems for Neurotrophic Factor Therapy

Neurotrophic factors, and glial cell line-derived neurotrophic factor (GDNF) in particular, have been regarded as one of the most promising molecules for PD. In this regard, several delivery systems have been designed focused on increasing GDNF stability and retention in the brain. Several studies have demonstrated the preclinical efficacy of microencapsulated GDNF in different PD animal models (rodents and monkeys) (Garbayo et al., 2009, 2011, 2016). In those studies, a single injection of microencapsulated GDNF achieved long term improvement of motor function and dopaminergic function restoration in parkinsonian monkeys with severe nigrostriatal degeneration. The injectable formulation localized GDNF within the putamen and prevented systemic off-target effects. GDNF showed trophic effects on the nigrostriatal pathway increasing striatal and nigral dopaminergic neurons. Moreover, microencapsulated GDNF did not elicit immunogenicity or cerebellar degeneration. This example demonstrates that MPs are an efficient vehicle for sustained GDNF delivery to the brain. In another approach, vascular endothelial growth factor (VEGF), a potent angiogenic factor with prosurvival effects in neuronal cultures, was combined with GDNF to enhance the action of the latter (Herrán et al., 2013). A pronounced tyrosine hydroxylase (TH) neuron recovery was observed in the SNc of parkinsonian rats. Later, a combinatorial strategy of NPs-containing GDNF and VEGF was locally applied in a partially lesioned rat PD model. Behavioral improvement was observed together with a significant enhancement of dopaminergic neurons both in the striatum and SNc, which corroborates previous work in GDNF and VEGF encapsulation. Interestingly, the synergistic effect of the therapeutic proteins allows dose reduction while still providing neurogenerative/neuroreparative effects (Hernandez et al., 2014). The direct nose to brain administration of GDNF-NPs is another promising trend. One of the most recent examples uses nanoencapsulated GDNF in lipid NPs (Hernando et al., 2018). In order to enhance the target NP delivery to the brain, the nanocarrier surface was modified with a cell-penetrating peptide named TAT. The formulation improved the nose to brain delivery of GDNF, thereby improving motor function recovery and GDNF neuroprotective effects when tested in a mouse PD model. An alternative approach to NPs is the use of liposomes. Uptake of the neurotrophic factor to the brain via intranasal delivery is enhanced when GDNF is encapsulated in a liposomal formulation (Migliore et al., 2014). In order to move forward with nose to brain delivery strategies greater formulation retention in the olfactory region needs to be achieved, together with better targeting of specific brain regions. Finally, another promising approach that has been undertaken for GDNF brain delivery is the use of nanoformulations able to cross the blood brain barrier through receptor-mediated-delivery. This strategy would allow non-invasive drug delivery to the brain. Based on this concept, neuroprotection has been observed after the intravenous administration of a GDNF nanoformulation (Huang et al., 2009). The NPs improved locomotor activity, reduced dopaminergic neuronal loss and enhanced monoamine neurotransmitter levels in parkinsonian rats. A remaining challenge is to target specific brain areas in order to avoid unwanted side effects.

Besides GDNF, other neurotrophic factor such as basic fibroblast growth factor (bFGF) have been evaluated. One example involves gelatin nanostructured lipid carriers encapsulating bFGF that can be targeted to the brain via nasal administration (Zhao et al., 2014). Overall, the nanoformulation stimulated dopaminergic function in surviving synapses and played a neuroprotective role in 6-OHDA hemiparkinsonian rats. A very recent study took advantage of the neuroprotective properties of Activin B, which was administered in a parkinsonian mice using a thermosensitive injectable HG (Li et al., 2016). The biomaterial allowed a sustained protein release over 5 weeks and contributed to substantial cellular protection and behavioral improvement.

### Drug Delivery Systems for Stem Cell Therapy

In recent years, stem cells have attracted considerable attention as regards achieving neuroprotection. However, cell therapy has been limited by the low engraftment of the administered cells. By applying a combination of biomaterials, cells and bioactive molecules, brain repair can be facilitated. In an early example, MPs loaded with neurotrophin-3 were used to retain injected adult stem cells in the striatum and to support cell viability and differentiation (Delcroix et al., 2011). When tested in a PD rat model, a potent behavioral recovery was observed together with nigrostriatal pathway protection/repair. Going a step further, BDNF-loaded MPs have been encapsulated in a HG embedded with mesenchymal stem cells for neural differentiation and secretome enhancement (Kandalam et al., 2017). This strategy not only provides neuroprotective BDNF but also stem cells that benefit from that environment by displaying neural commitment and an improved neuroprotective/reparative secretome. Likewise, HGs have also been used to improve dopaminergic progenitor survival and integration after transplantation. A report by T. Wang and co-workers pioneered the development of a composite scaffold made of nanofibers embedded within a xyloglucan HG. The biomaterial was further functionalized with GDNF to improve the niche surrounding the implanted cells (Wang T.Y. et al., 2016). The scaffold enhanced graft survival and striatal re-innervation. A similar strategy was followed by Adil MM, that determined the impact of a heparin/RGD functionalized hyaluronic acid HG on the survival of embryonic stem cell-derived dopaminergic neurons (Adil et al., 2017). These examples demonstrate the potential of biologically functionalized HGs to improve stem cell delivery. Beyond HGs, the use of NPs as a tool to optimize MSC therapeutics was underlined in a recent study by T. Chung and coworkers that successfully developed a dextran-coated iron oxide nanosystem to improve the rescuing effect of mesenchymal stem cells (Chung et al., 2018).

In addition to stem cell delivery, biomaterials can also be used to deliver mesenchymal stem cell secretome at the site of injury. By way of example, adipose mesenchymal stem cell secretome has been encapsulated in a biodegradable injectable HG that was able to increase the controlled release of the neuroprotective factors in a PD-relevant experimental context (Chierchia et al., 2017). NPs can also be used to modulate the subventricular neurogenic niche and boost endogenous brain repair mechanisms using microRNAs. Due to the short half-life and poor stability of these molecules, their efficient delivery into cells is a challenge. NPs can provide a shielded environment and controlled release. One example involves microRNA-124, a potent pro-neurogenic factor for neural stem cells which has been nanoencapsulated, demonstrating the feasibility of this approach as well as its efficacy in parkinsonian mice (Saraiva et al., 2016). The nanoformulation promoted not only neurogenesis but also the migration and maturation of new neurons in the lesioned striatum. Specifically, this example illustrates the potential of nanotechnology for improving not only the safety and efficacy of conventional drugs, but also the delivery of newer drugs based on microRNAs to the brain. Overall, these promising results suggest that biomaterials and drug delivery systems are a valid alternative to enhance stem cell neuroprotective properties. Further studies are needed for the advancement of this technology from preclinical studies to clinical trials.

### Nanomedicines for Antioxidant Delivery

Mitochondrial damage and oxidative stress have been proposed as the major contributing factors to PD pathogenesis. Accordingly, coenzyme Q10 has been considered a promising molecule in PD management due to its ability to enhance mitochondrial function. However, its efficacy has been hindered by insolubility, poor bioavailability and lack of brain penetration. In order to solve these issues, a nanomicellar coenzyme Q10 formulation able to stop, but not reverse, ongoing neurodegeneration has shown efficacy in a mouse PD model (Sikorska et al., 2014). Moreover, this neuroprotective treatment activates an astrocytic reaction suggesting that these cells played a significant role in neuron protection. In addition to coenzyme Q10, curcumin counteracts oxidative stress and mitochondrial dysfunction. However, its clinical efficacy has been limited by its poor aqueous solubility, rapid metabolism and inadequate tissue absorption. Piperine has been used as adjuvant to improve curcumin’s bioavailability. Thus, curcumin and piperine amalgamation seems beneficial. Moreover, nanomedicines could also help to enhance drug transport from blood to the brain. In one example, both therapeutics were loaded in a lipid-based nanoformulation blended with different surfactants and orally administered in a PD mouse model (Kundu et al., 2016). A higher density of nigral TH^+^ neurons was found in the animals treated with dual drug loaded NPs, demonstrating that the system was able to cross the blood brain barrier preventing dopaminergic neuronal degeneration. This may be due to the improved curcumine bioavailability and the synergistic effect exhibited by both drugs. Another strategy to detain oxidative stress and achieve neuroprotection is the use of nanoencapsulated resveratrol (da Rocha Lindner et al., 2015). The nanoformulation was able to attenuate MPTP-induced lipid peroxidation and prevent striatal TH protein decrease in parkinsonian mice. These findings suggest that resveratrol-loaded NPs are a promising nanomedical tool for PD.

### Nanomedicines That Interfere With α-syn Expression

Strategies that interfere with α-syn expression in neurons have also received widespread attention. One remarkable approach is the targeted gene therapy proposed by Niu et al. (2017) that has provided effective repair in a PD mice model using magnetic NPs loaded with shRNA plasmid for α-syn. Multifunctional magnetic NPs were effectively delivered through the blood brain barrier, prevented DA neuron degeneration as reflected by TH up-regulation and α-syn down-regulation and inhibited further apoptosis in the brain. Alternatively, suppression of α-syn overexpression has been demonstrated using gold NPs which could load plasmid DNA, cross the blood–brain barrier and target specific cells. For example, the group of Y. Guan achieved successful results in carrying pDNA into the neurons, and thus inhibiting dopaminergic neuron apoptosis (Hu et al., 2018). These approaches have the potential to suppress α-syn expression, providing a highly efficient treatment for PD.

### Focused Ultrasound

In the last few years, the use of focused ultrasound (FUS) therapies has been revolutionizing the treatment of neurological disorders. This non-invasive technique consists in the application of focused acoustic energy (ultrasound) on selected brain areas. The MR-guided FUS (MRgFUS) allowed computer calculated targeting and achieved high accuracy with real-time feedback on the effect of the treatment. The first studies using MRgFUS thalamotomy in patients with essential tremor showed a significant clinical reduction in hand tremor (Elias et al., 2016). In PD, MRgFUS is being explored as a way to non-invasively ablate the brain areas responsible for the motor features associated with the disease. In 2014, MRgFUS of the pallidothalamic tract was used in PD patients for the first time, with a significant clinical improvement (Magara et al., 2014). Subsequent studies using MRgFUS in the ventral intermediate thalamic nuclei (Vim) reported a clinically significant reduction in mean UPDRS scores post procedure in PD patients (Schlesinger et al., 2015). In a recent pilot study, MRgFUS unilateral subthalamotomy was reported to be well tolerated and to improve the motor features of noticeably asymmetric PD patients (Martínez-Fernández et al., 2018). The questions of the best target for treating PD symptoms and whether different targets should be chosen for different patients are currently unresolved. Other unanswered questions are the long-term durability of FUS ablation outcomes and the safety and feasibility of bilateral procedures. The possibility of this non-invasive approach, with its immediate and apparently permanent clinical outcome, makes this treatment suitable for an increasing number of patients who are either unable or unwilling to undergo DBS therapy. Large randomized controlled trials are necessary to validate these preliminary findings and to assess the potential use of ablative FUS therapy in the treatment of PD patients. Other applications of FUS that are under current research are the opening of the brain–blood barrier (BBB) or neuromodulation (Krishna et al., 2017). Low-Intensity Ultrasound decreased α-syn in PC12 cells (Karmacharya et al., 2017). And more recently, using a non-invasive approach by combining MRgFUS and intravenous microbubbles and a shRNA sequence targeting α-syn, immunoreactivity of this protein have been decreased in several regions such as hippocampus, SNpc, olfactory bulb, and dorsal motor nucleus (Xhima et al., 2018). This technology could be useful in the near future to alter the progression of LB pathology in combination with improved early diagnosis of the disease.

### Deep Brain Stimulation

Device-aided therapies, as levodopa–carbidopa infusion gel (LCIG), subcutaneous apomorphine pump infusion and deep brain stimulation (DBS), are essential tools in the treatment of advanced PD patients. During the last decade, evidence has been obtained regardless of safety, validity and efficacy in large prospective clinical studies (Antonini et al., 2018).

Deep brain stimulation is a surgical therapy that involves the implantation of one or more electrodes in specific regions of the brain. There is substantial and consistent evidence indicating that DBS of both STN and GPi improve motor fluctuations, dyskinesia and quality of life in advanced PD (Rodriguez-Oroz et al., 2005; Follett et al., 2010). Those benefits are maintained for more than 10 years (Zibetti et al., 2011). Additionally, DBS treatment has been evaluated in patients with relatively short disease duration providing better motor outcomes and quality of life compared to the control group receiving best medical treatment (Tinkhauser et al., 2018).

Deep brain stimulation has notably improved due to the development of new neurosurgery approaches (asleep surgery), devices (microelectrodes, directional electrodes), and programming and stimulation algorithms. Particularly relevant is the implementation of the directional electrodes, which leads to a segmented stimulation. They provide a more accurate therapeutic frame and potentially reduce the adverse effects related to DBS (Steigerwald et al., 2016).

The control of fluctuations could be improved and the adverse effects of DBS could be reduced by selective stimulation in a short-time window by using adaptive DBS (aDBS). Thus, aDBS is intended to personalize stimulation by recording local field potentials (LFP) directly from the stimulating electrode, which can only be activated when the LFP beta power exceeds a customized threshold. Therefore, it can modulate the stimulations according to the changes in the LFP beta power. aDBS seems to be more effective than conventional DBS in improving motor scores and controlling levodopa-induced dyskinesias. Further research over more extended time periods and larger cohorts are needed to ensure the benefit and efficacy of this novel strategy (Meidahl et al., 2017).

### Diagnosis and Clinical Assessment Devices

The use of new technology-based tools allows quantitative assessment of the motor function of PD patients. Sensors, video-assessment methods or mobile phone applications are some of the techniques that improve the sensitivity, accuracy and reproducibility of the evaluation of PD patients (Espay et al., 2016). Portable devices that include inertial measurement units (IMUs) measure the orientation, amplitude and frequency of movement, as well as the speed of the part of the body where they are located. IMUs are usually made up of accelerometers and gyroscopes, and occasionally magnetometers. IMUs situated in different parts of the patient’s body make a precise record of tremor, bradykinesia, dyskinesias and even gait patterns (Heldman et al., 2014). On the other hand, continual monitoring of the motor status in the domestic environment (regarding baseline motor status, motor fluctuations, and benefit of treatment, among other factors) is also possible by using these technology-based tools (Ossig et al., 2016). These new technology-based systems open up an unexpected range of specific and real-time data, thereby resulting in the prospect of (1) better diagnostic accuracy, (2) more sensitive monitoring of the motor and non-motor symptoms, and (3) more precise adjustments of medical therapies. However, their use is limited in routine clinical practice due to the heterogeneity of the studies, which limit the extrapolation of results, and the high cost of the devices (Sánchez-Ferro et al., 2016).

## Conclusion

In the future, population aging in developed countries will increase the burden of neurodegenerative diseases. In the case of PD, where treatment of symptoms needs to be patient-customized, balancing the control of symptoms, drug dose, presence of side effects and patient’s expectations, clinicians and researchers face a situation in which a synergy of medicine and research is urgently needed. In summary, 200 years after the publication of James Parkinson’s essay, our understanding of the disease has made remarkable progress and is still advancing, generating a considerable array of tools. Nowadays, fields such as functional genetics, novel molecular mechanisms, brain imaging and biomarker detection seem to be the major issues guiding our research strategies. Nevertheless, despite the progress made, improved early clinical diagnosis is still necessary and the disease lacks a cure. In this regard, research in drug delivery might provide safer and more effective treatments for PD. Years of research have revealed the need to take into account the role of environmental factors in addition to the genetics when studying PD progression. However, further research is needed to decipher the mechanisms by which this pathology spreads from cell to cell within the brain and from other organs to the central nervous system. Importantly, studies should also address early diagnosis (screening) tools, and more information is needed concerning the differential vulnerability of pathogenic factors affecting dopaminergic neurons.

## Author Contributions

NDR, AQ-V, EG, IC-C, RF-S, MM, IT-D, MB-P, and JB reviewed the literature, composed and wrote the manuscript. NDR, IT-D, and JB organized the paper. IC-C and RF-S prepared Table 1. AQ-V prepared Figure 1. EG prepared Figure 2.

## Conflict of Interest Statement

The authors declare that the research was conducted in the absence of any commercial or financial relationships that could be construed as a potential conflict of interest.

## References

[B1] Adil M. M. Vazin T. Ananthanarayanan B. Rodrigues G. M. C. Rao A. T. Kulkarni R. U. (2017). Engineered hydrogels increase the post-transplantation survival of encapsulated hESC-derived midbrain dopaminergic neurons. *Biomaterials* 136 1–11. 10.1016/j.biomaterials.2017.05.008 28505596

[B2] Anden N. E. Dahlstroem A. Fuxe K. Larsson K. (1965). Further evidence for the presence of nigro-neostriatal dopamine neurons in the rat. *Am. J. Anat.* 116 329–333. 10.1002/aja.1001160117 14283289

[B3] Antonini A. Moro E. Godeiro C. Reichmann H. (2018). Medical and surgical management of advanced Parkinson’s disease. *Mov. Disord.* 33 1–9. 10.1002/mds.27340 29570862

[B4] Barker R. A. Drouin-Ouellet J. Parmar M. (2015). Cell-based therapies for Parkinson disease—past insights and future potential. *Nat. Rev. Neurol.* 11 492–503. 10.1038/nrneurol.2015.123 26240036

[B5] Bergman H. Wichmann T. DeLong M. R. (1990). Reversal of experimental parkinsonism by lesions of the subthalamic nucleus. *Science* 249 1436–1438. 10.1126/science.2402638 2402638

[B6] Bertler A. Rosengren E. (1959). Occurrence and distribution of dopamine in brain and other tissues. *Experientia* 15 10–11. 10.1007/BF02157069 13619664

[B7] Blesa J. Phani S. Jackson-Lewis V. Przedborski S. (2012). Classic and new animal models of Parkinson’s disease. *J. Biomed. Biotechnol.* 2012:845618. 10.1155/2012/845618 22536024 PMC3321500

[B8] Blesa J. Trigo-Damas I. Dileone M. Lopez-Gonzalez del Rey N. Hernandez L. F. Obeso J. A. (2017). Compensatory mechanisms in Parkinson’s disease: circuits adaptations and role in disease modification. *Exp. Neurol.* 298(Pt B), 148–161. 10.1016/j.expneurol.2017.10.002 28987461

[B9] Blocq C. Marinescu G. (1893). Sur un cas de tremblement parkinsonien hémiplégique symptomatique d’une tumeur du pédoncle cérébral. *C. R. Cos. Biol.* 5 105–111.

[B10] Boeve B. F. Silber M. H. Ferman T. J. Lucas J. A. Parisi J. E. (2001). Association of REM sleep behavior disorder and neurodegenerative disease may reflect an underlying synucleinopathy. *Mov. Disord. Off. J. Mov. Disord. Soc.* 16 622–630. 10.1002/mds.1120 11481685

[B11] Bonifati V. (2010). Shaking the genome: new studies reveal genetic risk for Parkinson’s disease. *Lancet Neurol.* 9 136–138. 10.1016/S1474-4422(09)70363-1 20129160

[B12] Botta-Orfila T. Ezquerra M. Ríos J. Fernández-Santiago R. Cervantes S. Samaranch L. (2011). Lack of interaction of SNCA and MAPT genotypes in Parkinson’s disease. *Eur. J. Neurol.* 18:e32. 10.1111/j.1468-1331.2010.03245.x 21054681

[B13] Braak H. Del Tredici K. Rüb U. de Vos R. A. I. Jansen Steur E. N. H. Braak E. (2003). Staging of brain pathology related to sporadic Parkinson’s disease. *Neurobiol. Aging* 24 197–211. 10.1016/S0197-4580(02)00065-9 12498954

[B14] Bras J. Verloes A. Schneider S. A. Mole S. E. Guerreiro R. J. (2012). Mutation of the parkinsonism gene ATP13A2 causes neuronal ceroid-lipofuscinosis. *Hum. Mol. Genet.* 21 2646–2650. 10.1093/hmg/dds089 22388936 PMC3363329

[B15] Brissaud E. (1899). *Leçons sur les Maladies Nerveuses*, Vol. 2 Escondido, CA: Masson & Associates, Inc

[B16] Brockmann K. Schulte C. Hauser A. K. Lichtner P. Huber H. Maetzler W. (2013). SNCA: major genetic modifier of age at onset of Parkinson’s disease. *Mov. Disord.* 28 1217–1221. 10.1002/mds.25469 23674386

[B17] Buddhala C. Loftin S. K. Kuley B. M. Cairns N. J. Campbell M. C. Perlmutter J. S. (2015). Dopaminergic, serotonergic, and noradrenergic deficits in Parkinson disease. *Ann. Clin. Transl. Neurol.* 2 949–959. 10.1002/acn3.246 26478895 PMC4603378

[B18] Caffrey T. M. Wade-Martins R. (2007). Functional MAPT haplotypes: bridging the gap between genotype and neuropathology. *Neurobiol. Dis.* 27 1–10. 10.1016/j.nbd.2007.04.006 17555970 PMC2801069

[B19] Cardo L. F. Coto E. de Mena L. Ribacoba R. Lorenzo-Betancor O. Pastor P. (2012). A search for SNCA 3′ UTR variants identified SNP rs356165 as a determinant of disease risk and onset age in Parkinson’s disease. *J. Mol. Neurosci.* 47 425–430. 10.1007/s12031-011-9669-1 22076805

[B20] Carlsson A. (1959). The occurrence, distribution and physiological role of catecholamines in the nervous system. *Pharmacol. Rev.* 11 490–493. 13667431

[B21] Carlsson A. Lindqvist M. Magnusson T. (1957). 3,4-Dihydroxyphenylalanine and 5-hydroxytryptophan as reserpine antagonists. *Nature* 180:1200 10.1038/1801200a0 13483658

[B22] Carlsson A. Lindqvist M. Magnusson T. Waldeck B. (1958). On the presence of 3-hydroxytyramine in brain. *Science* 127:471 10.1126/science.127.3296.471 13529006

[B23] Charcot J. M. (1872). “LeÇons sur le maladies du système nerveux,” in *Oeuvres Complètes (Tome 1). Bureaux du Progrès Médical*, eds Delahaye A. Lecrosnier E. Paris: Bureaux du Progrès Médical 155–188.

[B24] Chartier-Harlin M.-C. Kachergus J. Roumier C. Mouroux V. Douay X. Lincoln S. (2004). α-synuclein locus duplication as a cause of familial Parkinson’s disease. *Lancet* 364 1167–1169. 10.1016/S0140-6736(04)17103-1 15451224

[B25] Chaudhuri K. R. Healy D. G. Schapira A. H. (2006). Non-motor symptoms of Parkinson’s disease: diagnosis and management. *Lancet Neurol.* 5 235–245. 10.1016/S1474-4422(06)70373-8 16488379

[B26] Chaudhuri K. R. Schapira A. H. V. (2009). Non-motor symptoms of Parkinson’s disease: dopaminergic pathophysiology and treatment. *Lancet Neurol.* 8 464–474. 10.1016/S1474-4422(09)70068-7 19375664

[B27] Chierchia A. Chirico N. Boeri L. Raimondi I. Riva G. A. Raimondi M. T. (2017). Secretome released from hydrogel-embedded adipose mesenchymal stem cells protects against the Parkinson’s disease related toxin 6-hydroxydopamine. *Eur. J. Pharm. Biopharm.* 121 113–120. 10.1016/j.ejpb.2017.09.014 28965958 PMC5656105

[B28] Chung T.-H. Hsu S.-C. Wu S.-H. Hsiao J.-K. Lin C.-P. Yao M. (2018). Dextran-coated iron oxide nanoparticle-improved therapeutic effects of human mesenchymal stem cells in a mouse model of Parkinson’s disease. *Nanoscale* 10 2998–3007. 10.1039/C7NR06976F 29372743

[B29] Cotzias G. C. (1968). L-Dopa for Parkinsonism. *N. Engl. J. Med.* 278:630 10.1056/NEJM196803142781127 5637779

[B30] Coupland K. G. Mellick G. D. Silburn P. A. Mather K. Armstrong N. J. Sachdev P. S. (2014). DNA methylation of the MAPT gene in Parkinson’s disease cohorts and modulation by vitamin E *in vitro*. *Mov. Disord.* 29 1606–1614. 10.1002/mds.25784 24375821 PMC4074263

[B31] da Rocha Lindner G. Bonfanti Santos D. Colle D. Gasnhar Moreira E. L. Daniel Prediger R. Farina M. (2015). Improved neuroprotective effects of resveratrol-loaded polysorbate 80-coated poly(lactide) nanoparticles in MPTP-induced Parkinsonism. *Nanomedicine* 10 1127–1138. 10.2217/nnm.14.165 25929569

[B32] Dahlstroem A. Fuxe K. (1964). Evidence for the existence of monoamine-containing neurons in the central nervous system. i. demonstration of monoamines in the cell bodies of brain stem neurons. *Acta Physiol. Scand. Suppl.* 232 1–55. 14229500

[B33] Davis G. C. Williams A. C. Markey S. P. Ebert M. H. Caine E. D. Reichert C. M. (1979). Chronic Parkinsonism secondary to intravenous injection of meperidine analogues. *Psychiatry Res.* 1 249–254. 10.1016/0165-1781(79)90006-4 298352

[B34] de Hemptinne C. Swann N. C. Ostrem J. L. Ryapolova-Webb E. S. San Luciano M. Galifianakis N. B. (2015). Therapeutic deep brain stimulation reduces cortical phase-amplitude coupling in Parkinson’s disease. *Nat. Neurosci.* 18 779–786. 10.1038/nn.3997 25867121 PMC4414895

[B35] Dehay B. Decressac M. Bourdenx M. Guadagnino I. Fernagut P.-O. Tamburrino A. (2016). Targeting α-synuclein: therapeutic options. *Mov. Disord.* 31 882–888. 10.1002/mds.26568 26926119

[B36] Delcroix G. J.-R. Garbayo E. Sindji L. Thomas O. Vanpouille-Box C. Schiller P. C. (2011). The therapeutic potential of human multipotent mesenchymal stromal cells combined with pharmacologically active microcarriers transplanted in hemi-parkinsonian rats. *Biomaterials* 32 1560–1573. 10.1016/j.biomaterials.2010.10.041 21074844

[B37] Di Fonzo A. Rohé C. F. Ferreira J. Chien H. F. Vacca L. Stocchi F. (2005). A frequent LRRK2 gene mutation associated with autosomal dominant Parkinson’s disease. *Lancet* 365 412–415. 10.1016/S0140-6736(05)17829-5 15680456

[B38] Edvardson S. Cinnamon Y. Ta-Shma A. Shaag A. Yim Y.-I. Zenvirt S. (2012). A deleterious mutation in DNAJC6 encoding the neuronal-specific clathrin-uncoating co-chaperone auxilin, is associated with juvenile parkinsonism. *PLoS One* 7:e36458. 10.1371/journal.pone.0036458 22563501 PMC3341348

[B39] Ehringer H. Hornykiewicz O. (1960). Distribution of noradrenaline and dopamine (3-hydroxytyramine) in the human brain and their behavior in diseases of the extrapyramidal system. *Klin. Wochenschr.* 38 1236–1239. 13726012 10.1007/BF01485901

[B40] Elias W. J. Lipsman N. Ondo W. G. Ghanouni P. Kim Y. G. Lee W. (2016). A randomized trial of focused ultrasound thalamotomy for essential tremor. *N. Engl. J. Med.* 375 730–739. 10.1056/NEJMoa1600159 27557301

[B41] Espay A. J. Bonato P. Nahab F. B. Maetzler W. Dean J. M. Klucken J. (2016). Technology in Parkinson’s disease: challenges and opportunities. *Mov. Disord.* 31 1272–1282. 10.1002/mds.26642 27125836 PMC5014594

[B42] Fahn S. (2003). Description of Parkinson’s disease as a clinical syndrome. *Ann. N. Y. Acad. Sci.* 991 1–14. 10.1111/j.1749-6632.2003.tb07458.x 12846969

[B43] Farrer M. J. (2006). Genetics of Parkinson disease: paradigm shifts and future prospects. *Nat. Rev. Genet.* 7 306–318. 10.1038/nrg1831 16543934

[B44] Farrer M. J. Stone J. T. Lin C. H. Dächsel J. C. Hulihan M. M. Haugarvoll K. (2007). Lrrk2 G2385R is an ancestral risk factor for Parkinson’s disease in Asia. *Parkinsonism Relat. Disord.* 13 89–92. 17222580 10.1016/j.parkreldis.2006.12.001

[B45] Fernández-Santiago R. Carballo-Carbajal I. Castellano G. Torrent R. Richaud Y. Sánchez-Danés A. (2015). Aberrant epigenome in iPSC-derived dopaminergic neurons from Parkinson’s disease patients. *EMBO Mol. Med.* 7 1529–1546. 10.15252/emmm.201505439 26516212 PMC4693505

[B46] Fernández-Santiago R. Garrido A. Infante J. González-Aramburu I. Sierra M. Fernández M. (2018). α-synuclein (SNCA) but not dynamin 3 (DNM3) influences age at onset of leucine-rich repeat kinase 2 (LRRK2) Parkinson’s disease in Spain. *Mov. Disord.* 33 637–641. 10.1002/mds.27295 29473656

[B47] Follett K. A. Weaver F. M. Stern M. Hur K. Harris C. L. Luo P. (2010). Pallidal versus subthalamic deep-brain stimulation for Parkinson’s Disease. *N. Engl. J. Med.* 362 2077–2091. 10.1056/NEJMoa0907083 20519680

[B48] Garbayo E. Ansorena E. Lana H. Carmona-Abellan M. D. Marcilla I. Lanciego J. L. (2016). Brain delivery of microencapsulated GDNF induces functional and structural recovery in parkinsonian monkeys. *Biomaterials* 110 11–23. 10.1016/j.biomaterials.2016.09.015 27697668

[B49] Garbayo E. Ansorena E. Lanciego J. L. Blanco-Prieto M. J. Aymerich M. S. (2011). Long-term neuroprotection and neurorestoration by glial cell-derived neurotrophic factor microspheres for the treatment of Parkinson’s disease. *Mov. Disord.* 26 1943–1947. 10.1002/mds.23793 21661048

[B50] Garbayo E. Montero-Menei C. N. Ansorena E. Lanciego J. L. Aymerich M. S. Blanco-Prieto M. J. (2009). Effective GDNF brain delivery using microspheres–a promising strategy for Parkinson’s disease. *J. Control. Release* 135 119–126. 10.1016/j.jconrel.2008.12.010 19154763

[B51] Golbe L. I. Lazzarini A. M. Spychala J. R. Johnson W. G. Stenroos E. S. Mark M. H. (2001). The tau A0 allele in Parkinson’s disease. *Mov. Disord.* 16 442–447. 10.1002/mds.1087 11391737

[B52] Gregory A. Westaway S. K. Holm I. E. Kotzbauer P. T. Hogarth P. Sonek S. (2008). Neurodegeneration associated with genetic defects in phospholipase A(2). *Neurology* 71 1402–1409. 10.1212/01.wnl.0000327094.67726.28 18799783 PMC2676964

[B53] Grosset D. Taurah L. Burn D. J. MacMahon D. Forbes A. Turner K. (2007). A multicentre longitudinal observational study of changes in self reported health status in people with Parkinson’s disease left untreated at diagnosis. *J. Neurol. Neurosurg. Psychiatry* 78 465–469. 10.1136/jnnp.2006.098327 17098846 PMC2117846

[B54] Gulati A. Forbes A. Stegie F. Kelly L. Clough C. Chaudhuri K. R. (2004). A clinical observational study of the pattern and occurrence of non-motor symptoms in PD disease ranging from early to advanced disease. *Mov. Disord.* 19:S406.

[B55] Halliday G. Lees A. Stern M. (2011). Milestones in Parkinson’s disease–clinical and pathologic features. *Mov. Disord. Off. J. Mov. Disord. Soc.* 26 1015–1021. 10.1002/mds.23669 21626546

[B56] Hammond C. Bergman H. Brown P. (2007). Pathological synchronization in Parkinson’s disease: networks, models and treatments. *Trends Neurosci.* 30 357–364. 10.1016/j.tins.2007.05.004 17532060

[B57] Healy D. G. Falchi M. O’Sullivan S. S. Bonifati V. Durr A. Bressman S. (2008). Phenotype, genotype, and worldwide genetic penetrance of LRRK2-associated Parkinson’s disease: a case-control study. *Lancet Neurol.* 7 583–590. 10.1016/S1474-4422(08)70117-0 18539534 PMC2832754

[B58] Heldman D. A. Espay A. J. LeWitt P. A. Giuffrida J. P. (2014). Clinician versus machine: reliability and responsiveness of motor endpoints in Parkinson’s disease. *Park. Relat. Disord.* 20 590–595. 10.1016/j.parkreldis.2014.02.022 24661464 PMC4028404

[B59] Hernandez D. G. Paisa C. Mcinerney-leo A. Jain S. Meyer-lindenberg A. Evans E. W. (2005). Clinical and positron emission tomography of parkinson ’ s disease caused by LRRK2. *Ann. Neurol.* 57 453–456. 10.1002/ana.20401 15732108

[B60] Hernandez R. M. Herran E. Requejo C. Ruiz-Ortega J. A. Aristieta A. Igartua M. (2014). Increased antiparkinson efficacy of the combined administration of VEGF- and GDNF-loaded nanospheres in a partial lesion model of Parkinson’s disease. *Int. J. Nanomed.* 9 2677–2687. 10.2147/IJN.S61940 24920904 PMC4043720

[B61] Hernando S. Herran E. Figueiro-Silva J. Pedraz J. L. Igartua M. Carro E. (2018). Intranasal administration of tat-conjugated lipid nanocarriers loading GDNF for Parkinson’s disease. *Mol. Neurobiol.* 55 145–155. 10.1007/s12035-017-0728-7 28866799

[B62] Herrán E. Ruiz-Ortega J. Á Aristieta A. Igartua M. Requejo C. Lafuente J. V. (2013). In vivo administration of VEGF- and GDNF-releasing biodegradable polymeric microspheres in a severe lesion model of Parkinson’s disease. *Eur. J. Pharm. Biopharm.* 85 1183–1190. 10.1016/j.ejpb.2013.03.034 23639739

[B63] Hornykiewicz O. (2002). L-DOPA: from a biologically inactive amino acid to a successful therapeutic agent. *Amino Acids* 23 65–70. 10.1007/s00726-001-0111-9 12373520

[B64] Hu K. Chen X. Chen W. Zhang L. Li J. Ye J. (2018). Neuroprotective effect of gold nanoparticles composites in Parkinson’s disease model. *Nanomedicine* 14 1123–1136. 10.1016/j.nano.2018.01.020 29474924

[B65] Huang R. Han L. Li J. Ren F. Ke W. Jiang C. (2009). Neuroprotection in a 6-hydroxydopamine-lesioned Parkinson model using lactoferrin-modified nanoparticles. *J. Gene Med.* 11 754–763. 10.1002/jgm.1361 19554623

[B66] Ibáñez P. Bonnet A.-M. Débarges B. Lohmann E. Tison F. Agid Y. (2004). Causal relation between α-synuclein locus duplication as a cause of familial Parkinson’s disease. *Lancet* 364 1169–1171. 10.1016/S0140-6736(04)17104-3 15451225

[B67] Iranzo A. Stockner H. Serradell M. Seppi K. Valldeoriola F. Frauscher B. (2014). Five-year follow-up of substantia nigra echogenicity in idiopathic REM sleep behavior disorder. *Mov. Disord.* 29 1774–1780. 10.1002/mds.26055 25384461

[B68] Jellinger K. A. (2012). Neuropathology of sporadic Parkinson’s disease: evaluation and changes of concepts. *Mov. Disord. Off. J. Mov. Disord. Soc.* 27 8–30. 10.1002/mds.23795 22081500

[B69] Kachergus J. Mata I. F. Hulihan M. Taylor J. P. Lincoln S. Aasly J. (2005). Identification of a novel LRRK2 mutation linked to autosomal dominant parkinsonism: evidence of a common founder across European populations. *Am. J. Hum. Genet.* 76 672–680. 10.1086/429256 15726496 PMC1199304

[B70] Kandalam S. Sindji L. Delcroix G. J.-R. Violet F. Garric X. André E. M. (2017). Pharmacologically active microcarriers delivering BDNF within a hydrogel: Novel strategy for human bone marrow-derived stem cells neural/neuronal differentiation guidance and therapeutic secretome enhancement. *Acta Biomater.* 49 167–180. 10.1016/j.actbio.2016.11.030 27865962

[B71] Karmacharya M. B. Hada B. Park S. R. Choi B. H. (2017). Low-intensity ultrasound decreases α-synuclein aggregation via attenuation of mitochondrial reactive oxygen species in MPP(+)-treated PC12 cells. *Mol. Neurobiol.* 54 6235–6244. 10.1007/s12035-016-0104-z 27714630

[B72] Kim J. Inoue K. Ishii J. Vanti W. B. Voronov S. V. Murchison E. (2007). A MicroRNA feedback circuit in midbrain dopamine neurons. *Science* 317 1220–1224. 10.1126/science.1140481 17761882 PMC2782470

[B73] Ko W. K. D. Bezard E. (2017). Experimental animal models of Parkinson’s disease: a transition from assessing symptomatology to α-synuclein targeted disease modification. *Exp. Neurol.* 298(Pt B), 172–179. 10.1016/j.expneurol.2017.07.020 28764902

[B74] Koprich J. B. Kalia L. V. Brotchie J. M. (2017). Animal models of α-synucleinopathy for Parkinson disease drug development. *Nat. Rev. Neurosci.* 18 515–529. 10.1038/nrn.2017.75 28747776

[B75] Kösel S. Grasbon-Frodl E. M. Mautsch U. Egensperger R. von Eitzen U. Frishman D. (1998). Novel mutations of mitochondrial complex I in pathologically proven Parkinson disease. *Neurogenetics* 1 197–204. 10.1007/s100480050029 10737123

[B76] Krishna V. Sammartino F. Rezai A. (2017). A review of the current therapies, challenges, and future directions of transcranial focused ultrasound technology: advances in diagnosis and treatment. *JAMA Neurol.* 75 246–254. 10.1001/jamaneurol.2017.3129 29228074

[B77] Krüger R. Kuhn W. Müller T. Woitalla D. Graeber M. Kösel S. (1998). Ala30Pro mutation in the gene encoding alpha-synuclein in Parkinson’s disease. *Nat. Genet.* 18 106–108. 10.1038/ng0298-106 9462735

[B78] Kundu P. Das M. Tripathy K. Sahoo S. K. (2016). Delivery of dual drug loaded lipid based nanoparticles across the blood-brain barrier impart enhanced neuroprotection in a rotenone induced mouse model of parkinson’s disease. *ACS Chem. Neurosci.* 7 1658–1670. 10.1021/acschemneuro.6b00207 27642670

[B79] Langston J. W. (2017). The MPTP story. *J. Parkinsons Dis.* 7 S11–S19. 10.3233/JPD-179006 28282815 PMC5345642

[B80] Langston J. W. Ballard P. Tetrud J. W. Irwin I. (1983). Chronic parkinsonism in humans due to a product of meperidine-analog synthesis. *Science* 219 979–980. 10.1126/science.6823561 6823561

[B81] Lázaro D. F. Pavlou M. A. S. Outeiro T. F. (2017). Cellular models as tools for the study of the role of alpha-synuclein in Parkinson’s disease. *Exp. Neurol.* 298(Pt B), 162–171. 10.1016/j.expneurol.2017.05.007 28526239

[B82] Lesage S. Anheim M. Letournel F. Bousset L. Honoré A. Rozas N. (2013). G51D α-synuclein mutation causes a novel Parkinsonian-pyramidal syndrome. *Ann. Neurol.* 73 459–471. 10.1002/ana.23894 23526723

[B83] Lesage S. Drouet V. Majounie E. Deramecourt V. Jacoupy M. Nicolas A. (2016). Loss of VPS13C function in autosomal-recessive parkinsonism causes mitochondrial dysfunction and Increases PINK1/Parkin-dependent mitophagy. *Am. J. Hum. Genet.* 98 500–513. 10.1016/j.ajhg.2016.01.014 26942284 PMC4800038

[B84] Lewy F. (1912). Zur pathologischen anatomie der paralysis agitans. *Dtsch. Z. Nervenheilkd.* 50 50–55.

[B85] Li J. Darabi M. Gu J. Shi J. Xue J. Huang L. (2016). A drug delivery hydrogel system based on activin B for Parkinson’s disease. *Biomaterials* 102 72–86. 10.1016/j.biomaterials.2016.06.016 27322960

[B86] Lill C. M. (2016). Genetics of Parkinson’s disease. *Mol. Cell. Probes* 30 386–396. 10.1016/j.mcp.2016.11.001 27818248

[B87] Lu C.-S. Wu-Chou Y. H. van Doeselaar M. Simons E. J. Chang H.-C. Breedveld G. J. (2008). The LRRK2 Arg1628Pro variant is a risk factor for Parkinson’s disease in the Chinese population. *Neurogenetics* 9 271–276. 10.1007/s10048-008-0140-6 18716801

[B88] Magara A. Bühler R. Moser D. Kowalski M. Pourtehrani P. Jeanmonod D. (2014). First experience with MR-guided focused ultrasound in the treatment of Parkinson’s disease. *J. Ther. Ultrasound* 2:11. 10.1186/2050-5736-2-11 25512869 PMC4266014

[B89] Marder K. Wang Y. Alcalay R. N. Mejia-Santana H. Tang M. X. Lee A. (2015). Age-specific penetrance of LRRK2 G2019S in the michael j fox ashkenazi jewish lrrk2 consortium. *Neurology* 85 89–95. 10.1212/WNL.0000000000001708 26062626 PMC4501942

[B90] Marmion D. J. Kordower J. H. (2018). α-Synuclein nonhuman primate models of Parkinson’s disease. *J. Neural Transm.* 125 385–400. 10.1007/s00702-017-1720-0 28434076

[B91] Marras C. Alcalay R. N. Caspell-Garcia C. Coffey C. Chan P. Duda J. E. (2016). Motor and nonmotor heterogeneity of LRRK2-related and idiopathic Parkinson’s disease. *Mov. Disord.* 31 1192–1202. 10.1002/mds.26614 27091104

[B92] Martínez-Fernández R. Rodríguez-Rojas R. del Álamo M. Hernández-Fernández F. Pineda-Pardo J. A. Dileone M. (2018). Focused ultrasound subthalamotomy in patients with asymmetric Parkinson’s disease: a pilot study. *Lancet Neurol.* 17 54–63. 10.1016/S1474-4422(17)30403-9 29203153

[B93] Masliah E. Dumaop W. Galasko D. Desplats P. (2013). Distinctive patterns of DNA methylation associated with Parkinson disease: identification of concordant epigenetic changes in brain and peripheral blood leukocytes. *Epigenetics* 8 1030–1038. 10.4161/epi.25865 23907097 PMC3891683

[B94] Meidahl A. C. Tinkhauser G. Herz D. M. Cagnan H. Debarros J. Brown P. (2017). Adaptive deep brain stimulation for movement disorders: the long road to clinical therapy. *Mov. Disord.* 32 810–819. 10.1002/mds.27022 28597557 PMC5482397

[B95] Meyers R. (1942). The modification of alternating tremors, rigidity and festination by surgery of the basal ganglia. *Res. Publ. Assoc. Res. Nerv. Ment. Dis.* 21 602–665.

[B96] Migliore M. M. Ortiz R. Dye S. Campbell R. B. Amiji M. M. Waszczak B. L. (2014). Neurotrophic and neuroprotective efficacy of intranasal GDNF in a rat model of Parkinson’s disease. *Neuroscience* 274 11–23. 10.1016/j.neuroscience.2014.05.019 24845869

[B97] Montagu K. A. (1957). Catechol compounds in rat tissues and in brains of different animals. *Nature* 180 244–245. 10.1038/180244a0 13451690

[B98] Morissette M. Di Paolo T. (2018). Non-human primate models of PD to test novel therapies. *J. Neural Transm.* 125 291–324. 10.1007/s00702-017-1722-y 28391443

[B99] Nalls M. A. Pankratz N. Lill C. M. Do C. B. Hernandez D. G. Saad M. (2014). Large-scale meta-analysis of genome-wide association data identifies six new risk loci for Parkinson’s disease. *Nat. Genet.* 46 989–993. 10.1038/ng.3043 25064009 PMC4146673

[B100] Niu S. Zhang L.-K. Zhang L. Zhuang S. Zhan X. Chen W.-Y. (2017). Inhibition by multifunctional magnetic nanoparticles loaded with alpha-synuclein rnai plasmid in a Parkinson’s Disease Model. *Theranostics* 7 344–356. 10.7150/thno.16562 28042339 PMC5197069

[B101] Ossig C. Gandor F. Fauser M. Bosredon C. Churilov L. Reichmann H. (2016). Correlation of quantitative motor state assessment using a kinetograph and patient diaries in advanced PD: data from an observational study. *PLoS One* 11:e0161559. 10.1371/journal.pone.0161559 27556806 PMC4996447

[B102] O’Sullivan S. S. Williams D. R. Gallagher D. A. Massey L. A. Silveira-Moriyama L. Lees A. J. (2008). Nonmotor symptoms as presenting complaints in Parkinson’s disease: a clinicopathological study. *Mov. Disord. Off. J. Mov. Disord. Soc.* 23 101–106. 10.1002/mds.21813 17994582

[B103] Paisan-Ruiz C. Jain S. Evans E. W. Gilks W. P. Simon J. van der Brug M. (2004). Cloning of the gene containing mutations that cause PARK8-linked Parkinson’s disease. *Neuron* 44 595–600. 10.1016/j.neuron.2004.10.023 15541308

[B104] Park G. Tan J. Garcia G. Kang Y. Salvesen G. Zhang Z. (2016). Regulation of histone acetylation by autophagy in parkinson disease. *J. Biol. Chem.* 291 3531–3540. 10.1074/jbc.M115.675488 26699403 PMC4751393

[B105] Parkinson J. (1817). An essay on the shaking palsy. *J. Neuropsychiatry Clin. Neurosci.* 14 223–236. 10.1176/jnp.14.2.223 11983801

[B106] Pastor P. Ezquerra M. Munoz E. Martí M. J. Blesa R. Tolosa E. (2000). Significant association between the tau gene A0/A0 genotype, and Parkinson’s disease. *Ann Neurol.* 47 242–245. 10.1002/1531-8249(200002)47:2<242::AID-ANA16>3.0.CO;2-L 10665497

[B107] Pihlstrom L. Berge V. Rengmark A. Toft M. (2015). Parkinson’s disease correlates with promoter methylation in the alpha-synuclein gene. *Mov. Disord. J. Mov. Disord. Soc.* 30 577–580. 10.1002/mds.26073 25545759

[B108] Politis M. Wu K. Molloy S. Bain P. G. Chaudhuri K. R. Piccini P. (2010). Parkinson’s disease symptoms: the patient’s perspective. *Mov. Disord. Off. J. Mov. Disord. Soc.* 25 1646–1651. 10.1002/mds.23135 20629164

[B109] Polymeropoulos M. H. Lavedan C. Leroy E. Ide S. E. Dehejia A. Dutra A. (1997). Mutation in the alpha-synuclein gene identified in families with Parkinson’s disease. *Science* 276 2045–2047. 10.1126/science.276.5321.2045 9197268

[B110] Pont-Sunyer C. Tolosa E. Caspell-Garcia C. Coffey C. Alcalay R. N. Chan P. (2017). The prodromal phase of leucine-rich repeat kinase 2-associated Parkinson disease: clinical and imaging Studies. *Mov. Disord.* 32 726–738. 10.1002/mds.26964 28370517

[B111] Postuma R. B. Berg D. (2016). Advances in markers of prodromal Parkinson disease. *Nat. Rev. Neurol.* 12 622–634. 10.1038/nrneurol.2016.152 27786242

[B112] Postuma R. B. Gagnon J.-F. Pelletier A. Montplaisir J. Y. (2017). Insomnia and somnolence in idiopathic RBD: a prospective cohort study. *NPJ Park. Dis.* 3:9. 10.1038/s41531-017-0011-7 28649609 PMC5445588

[B113] Proukakis C. Dudzik C. G. Brier T. MacKay D. S. Cooper J. M. Millhauser G. L. (2013). A novel α-synuclein missense mutation in Parkinson disease. *Neurology* 80 1062–1064. 10.1212/WNL.0b013e31828727ba 23427326 PMC3653201

[B114] Rodríguez-Nogales C. Garbayo E. Carmona-Abellán M. M. Luquin M. R. Blanco-Prieto M. J. (2016). Brain aging and Parkinson’s disease: new therapeutic approaches using drug delivery systems. *Maturitas* 84 25–31. 10.1016/j.maturitas.2015.11.009 26653838

[B115] Rodriguez-Oroz M. C. Obeso J. A. Lang A. E. Houeto J. L. Pollak P. Rehncrona S. (2005). Bilateral deep brain stimulation in Parkinson’s disease: a multicentre study with 4 years follow-up. *Brain* 128 2240–2249. 10.1093/brain/awh571 15975946

[B116] Rosin B. Slovik M. Mitelman R. Rivlin-Etzion M. Haber S. N. Israel Z. (2011). Closed-loop deep brain stimulation is superior in ameliorating parkinsonism. *Neuron* 72 370–384. 10.1016/j.neuron.2011.08.023 22017994

[B117] Sakakibara R. Tateno F. Kishi M. Tsuyusaki Y. Terada H. Inaoka T. (2014). MIBG myocardial scintigraphy in pre-motor Parkinson’s disease: a review. *Parkinsonism Relat. Disord.* 20 267–273. 10.1016/j.parkreldis.2013.11.001 24332912

[B118] Samaranch L. Lorenzo-Betancor O. Arbelo J. M. Ferrer I. Lorenzo E. Irigoyen J. (2010). PINK1-linked parkinsonism is associated with Lewy body pathology. *Brain* 133 1128–1142. 10.1093/brain/awq051 20356854

[B119] Sampson T. R. Debelius J. W. Thron T. Janssen S. Shastri G. G. Ilhan Z. E. (2016). Gut microbiota regulate motor deficits and neuroinflammation in a model of parkinson’s disease. *Cell* 167 1469.e12–1480.e12. 10.1016/j.cell.2016.11.018 27912057 PMC5718049

[B120] Sánchez-Ferro Á Elshehabi M. Godinho C. Salkovic D. Hobert M. A. Domingos J. (2016). New methods for the assessment of Parkinson’s disease (2005 to 2015): a systematic review. *Mov. Disord.* 31 1283–1292. 10.1002/mds.26723 27430969

[B121] Sano H. (2000). Biochemistry of the extrapyramidal system shinkei kennkyu no shinpo, advances in neurological sciences. (ISSN 0001-8724) tokyo, october 1960;5:42-48. *Park. Relat. Disord.* 6 3–6. 10.1016/S1353-8020(99)00046-2 18591145

[B122] Sano I. Gamo T. Kakimoto Y. Taniguchi K. Takesada M. Nishinuma K. (1959). Distribution of catechol compounds in human brain. *Biochim. Biophys. Acta* 32 586–587. 10.1016/0006-3002(59)90652-3 14441532

[B123] Saraiva C. Paiva J. Santos T. Ferreira L. Bernardino L. (2016). MicroRNA-124 loaded nanoparticles enhance brain repair in Parkinson’s disease. *J. Control. Release* 235 291–305. 10.1016/j.jconrel.2016.06.005 27269730

[B124] Schapira A. H. (1993). Mitochondrial complex I deficiency in Parkinson’s disease. *Adv. Neurol.* 60 288–291. 10.1016/j.ymgme.2011.11.193 8420145

[B125] Schlesinger I. Eran A. Sinai A. Erikh I. Nassar M. Goldsher D. (2015). MRI guided focused ultrasound thalamotomy for moderate-to-severe tremor in Parkinson’s disease. *Parkinsons. Dis.* 2015:219149. 10.1155/2015/219149 26421209 PMC4572440

[B126] Schneider S. A. Alcalay R. N. (2017). Neuropathology of genetic synucleinopathies with parkinsonism: review of the literature. *Mov. Disord.* 32 1504–1523. 10.1002/mds.27193 29124790 PMC5726430

[B127] Seppi K. Weintraub D. Coelho M. Perez-Lloret S. Fox S. H. Katzenschlager R. (2011). The Movement disorder society evidence-based medicine review update: treatments for the non-motor symptoms of Parkinson’s disease. *Mov. Disord. Off. J. Mov. Disord. Soc.* 26(Suppl. 3), S42–S80. 10.1002/mds.23884 22021174 PMC4020145

[B128] Shojaee S. Sina F. Banihosseini S. S. Kazemi M. H. Kalhor R. Shahidi G.-A. (2008). Genome-wide linkage analysis of a parkinsonian-pyramidal syndrome pedigree by 500 K SNP arrays. *Am. J. Hum. Genet.* 82 1375–1384. 10.1016/j.ajhg.2008.05.005 18513678 PMC2427312

[B129] Shulman L. Taback R. Rabinstein A. Weiner W. (2002). Non-recognition of depression and other non-motor symptoms in Parkinson’s disease. *Park. Relat. Disord.* 8 193–197. 10.1016/S1353-8020(01)00015-3 12039431

[B130] Sikorska M. Lanthier P. Miller H. Beyers M. Sodja C. Zurakowski B. (2014). Nanomicellar formulation of coenzyme Q10 (Ubisol-Q10) effectively blocks ongoing neurodegeneration in the mouse 1-methyl-4-phenyl-1,2,3,6-tetrahydropyridine model: potential use as an adjuvant treatment in Parkinson’s disease. *Neurobiol. Aging* 35 2329–2346. 10.1016/j.neurobiolaging.2014.03.032 24775711 PMC4892899

[B131] Simón-Sánchez J. Schulte C. Bras J. M. Sharma M. Gibbs J. R. Berg D. (2009). Genome-wide association study reveals genetic risk underlying Parkinson’s disease. *Nat. Genet.* 41 1308–1312. 10.1038/ng.487 19915575 PMC2787725

[B132] Singleton A. B. Farrer M. Johnson J. Singleton A. Hague S. Kachergus J. (2003). alpha-synuclein locus triplication causes Parkinson’s disease. *Science* 302:841. 10.1126/science.1090278 14593171

[B133] Singleton A. B. Farrer M. J. Bonifati V. (2013). The genetics of Parkinson’s disease: progress and therapeutic implications. *Mov. Disord.* 28 14–23. 10.1002/mds.25249 23389780 PMC3578399

[B134] Smith Y. Wichmann T. Factor S. A. DeLong M. R. (2012). Parkinson’s disease therapeutics: new developments and challenges since the introduction of levodopa. *Neuropsychopharmacology* 37 213–246. 10.1038/npp.2011.212 21956442 PMC3238085

[B135] Spillantini M. G. Crowther R. A. Jakes R. Hasegawa M. Goedert M. (1998). alpha-synuclein in filamentous inclusions of lewy bodies from parkinson’s disease and dementia with lewy bodies. *Proc. Natl. Acad. Sci. U.S.A.* 95 6469–6473. 10.1073/pnas.95.11.6469 9600990 PMC27806

[B136] Spillantini M. G. Schmidt M. L. Lee V. M. Trojanowski J. Q. Jakes R. Goedert M. (1997). Alpha-synuclein in Lewy bodies. *Nature* 388 839–840. 10.1038/42166 9278044

[B137] Steigerwald F. Müller L. Johannes S. Matthies C. Volkmann J. (2016). Directional deep brain stimulation of the subthalamic nucleus: a pilot study using a novel neurostimulation device. *Mov. Disord.* 31 1240–1243. 10.1002/mds.26669 27241197 PMC5089579

[B138] Strauss K. M. Martins L. M. Plun-Favreau H. Marx F. P. Kautzmann S. Berg D. (2005). Loss of function mutations in the gene encoding Omi/HtrA2 in Parkinson’s disease. *Hum. Mol. Genet.* 14 2099–2111. 10.1093/hmg/ddi215 15961413

[B139] Taipa R. Pereira C. Reis I. Alonso I. Bastos-Lima A. Melo-Pires M. (2016). DJ-1 linked parkinsonism (PARK7) is associated with Lewy body pathology. *Brain* 139 1680–1687. 10.1093/brain/aww080 27085187

[B140] Thaler A. Gurevich T. Bar Shira A. Gana Weisz M. Ash E. Shiner T. (2017). A “dose” effect of mutations in the GBA gene on Parkinson’s disease phenotype. *Park. Relat. Disord.* 36 47–51. 10.1016/j.parkreldis.2016.12.014 28012950

[B141] Thobois S. Ardouin C. Schmitt E. Lhommée E. Klinger H. Xie J. (2010). Behavioral disorders in Parkinson’s disease: from pathophysiology to the mastery of dopaminergic treatment. *Rev. Neurol.* 166 816–821. 10.1016/j.neurol.2010.07.006 20739041

[B142] Tinkhauser G. Pogosyan A. Debove I. Nowacki A. Shah S. A. Seidel K. (2018). Directional local field potentials: a tool to optimize deep brain stimulation. *Mov. Disord.* 33 159–164. 10.1002/mds.27215 29150884 PMC5768242

[B143] Tolosa E. Gaig C. Santamaria J. Compta Y. (2009). Diagnosis and the premotor phase of Parkinson disease. *Neurology* 72 S12–S20. 10.1212/WNL.0b013e318198db11 19221308

[B144] Trétiakoff C. D. (1919). *Contribution à L’étude De L’anatomie Pathologique Du Locus Niger De Soemmering Avec Quelques Deductions Relatives A La Pathogenie Des Troubles Du Tonus Musculaire Et De La Maladie De Parkinson*. Paris: Universtié de Paris.

[B145] Trigo-Damas I. Del Rey N. L.-G. Blesa J. (2018). Novel models for Parkinson’s disease and their impact on future drug discovery. *Expert Opin. Drug Discov.* 13 229–239. 10.1080/17460441.2018.1428556 29363335

[B146] Trinh J. Gustavsson E. K. Vilariño-Güell C. Bortnick S. Latourelle J. McKenzie M. B. (2016). DNM3 and genetic modifiers of age of onset in LRRK2 Gly2019Ser parkinsonism: a genome-wide linkage and association study. *Lancet Neurol.* 15 1248–1256. 10.1016/S1474-4422(16)30203-4 27692902

[B147] van der Walt J. M. Nicodemus K. K. Martin E. R. Scott W. K. Nance M. A. Watts R. L. (2003). Mitochondrial polymorphisms significantly reduce the risk of Parkinson disease. *Am. J. Hum. Genet.* 72 804–811. 10.1086/373937 12618962 PMC1180345

[B148] Vilariño-Güell C. Wider C. Ross O. A. Dachsel J. C. Kachergus J. M. Lincoln S. J. (2011). VPS35 mutations in Parkinson disease. *Am. J. Hum. Genet.* 89 162–167. 10.1016/j.ajhg.2011.06.001 21763482 PMC3135796

[B149] Voon V. Fernagut P.-O. O. Wickens J. Baunez C. Rodriguez M. Pavon N. (2009). Chronic dopaminergic stimulation in Parkinson’s disease: from dyskinesias to impulse control disorders. *Lancet Neurol.* 8 1140–1149. 10.1016/S1474-4422(09)70287-X 19909912

[B150] Voon V. Mehta A. R. Hallett M. (2011). Impulse control disorders in Parkinson’s disease. *Curr. Opin. Neurol.* 24 324–330. 10.1097/WCO.0b013e3283489687 21725242 PMC3154756

[B151] Wang G. Huang Y. Chen W. Chen S. Wang Y. Xiao Q. (2016). Variants in the SNCA gene associate with motor progression while variants in the MAPT gene associate with the severity of Parkinson’s disease. *Park. Relat. Disord.* 24 89–94. 10.1016/j.parkreldis.2015.12.018 26776090

[B152] Wang T. Y. Bruggeman K. F. Kauhausen J. A. Rodriguez A. L. Nisbet D. R. Parish C. L. (2016). Functionalized composite scaffolds improve the engraftment of transplanted dopaminergic progenitors in a mouse model of Parkinson’s disease. *Biomaterials* 74 89–98. 10.1016/j.biomaterials.2015.09.039 26454047

[B153] Weintraub D. (2009). Dopamine and impulse control disorders in Parkinson’s disease. *Ann. Neurol.* 64 S93–S100. 10.1002/ana.21454 19127573 PMC3530139

[B154] Weintraub D. Koester J. Potenza M. N. Siderowf A. D. Stacy M. Voon V. (2010). Impulse control disorders in Parkinson disease: a cross-sectional study of 3090 patients. *Arch. Neurol.* 67 589–595. 10.1001/archneurol.2010.65 20457959

[B155] Witt K. Daniels C. Reiff J. Krack P. Volkmann J. Pinsker M. O. (2008). Neuropsychological and psychiatric changes after deep brain stimulation for Parkinson’s disease: a randomised, multicentre study. *Lancet Neurol.* 7 605–614. 10.1016/S1474-4422(08)70114-5 18538636

[B156] Wolters E. C. (2009). Non-motor extranigral signs and symptoms in Parkinson’s disease. *Parkinsonism Relat. Disord.* 15(Suppl. 3), S6–S12. 10.1016/S1353-8020(09)70770-9 20083010

[B157] Xhima K. Nabbouh F. Hynynen K. Aubert I. Tandon A. (2018). Noninvasive delivery of an α-synuclein gene silencing vector with magnetic resonance-guided focused ultrasound. *Mov. Disord.* 33 1567–1579. 10.1002/mds.101 30264465 PMC6282171

[B158] Zarranz J. J. Alegre J. Gómez-Esteban J. C. Lezcano E. Ros R. Ampuero I. (2004). The new mutation, E46K, of alpha-synuclein causes Parkinson and Lewy body dementia. *Ann. Neurol.* 55 164–173. 10.1002/ana.10795 14755719

[B159] Zesiewicz T. A. Sullivan K. L. Arnulf I. Chaudhuri K. R. Morgan J. C. Gronseth G. S. (2010). Practice parameter: treatment of nonmotor symptoms of parkinson disease: report of the quality standards subcommittee of the american academy of neurology. *Neurology* 74 924–931. 10.1212/WNL.0b013e3181d55f24 20231670

[B160] Zhao Y.-Z. Li X. Lu C.-T. Lin M. Chen L.-J. Xiang Q. (2014). Gelatin nanostructured lipid carriers-mediated intranasal delivery of basic fibroblast growth factor enhances functional recovery in hemiparkinsonian rats. *Nanomed. Nanotechnol. Biol. Med.* 10 755–764. 10.1016/j.nano.2013.10.009 24200526

[B161] Zibetti M. Merola A. Rizzi L. Ricchi V. Angrisano S. Azzaro C. (2011). Beyond nine years of continuous subthalamic nucleus deep brain stimulation in Parkinson’s disease. *Mov. Disord.* 26 2327–2334. 10.1002/mds.23903 22012750

[B162] Zimprich A. Benet-Pagès A. Struhal W. Graf E. Eck S. H. Offman M. N. (2011). A mutation in VPS35, encoding a subunit of the retromer complex, causes late-onset Parkinson disease. *Am. J. Hum. Genet.* 89 168–175. 10.1016/j.ajhg.2011.06.008 21763483 PMC3135812

[B163] Zimprich A. Biskup S. Leitner P. Lichtner P. Farrer M. Lincoln S. (2004). Mutations in LRRK2 cause autosomal-dominant parkinsonism with pleomorphic pathology. *Neuron* 44 601–607. 10.1016/j.neuron.2004.11.005 15541309

